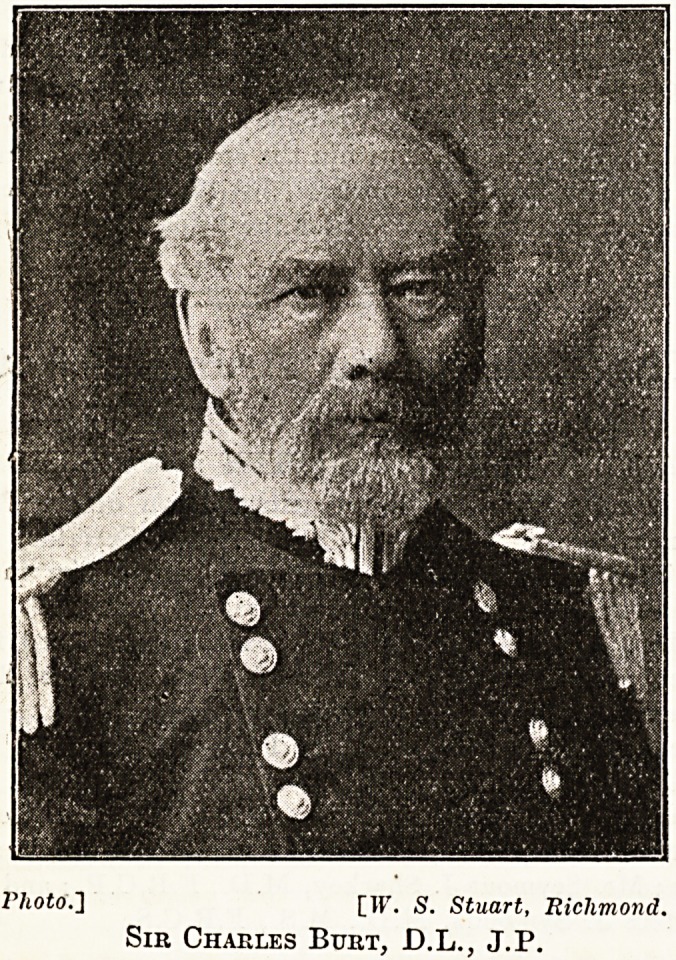# The Late Sir Charles Burt

**Published:** 1913-03-22

**Authors:** 


### The Late Sir Charles Burt, D.L., J.P.

Sir Charles Burt during his working years was one of the ablest and most trusted of City solicitors, having been associated with the firm of Messrs. Bircham and Co., of which he became the senior partner before his retirement in 1889. We had the pleasure of being associated with him in the City for many years, and can therefore estimate his great qualities as a solicitor and his excellence as a man of business. On his retirement, Mr. Burt, as he then was, went to Richmond, and identified himself closely with local affairs there, in which he took an active part. Sir Charles Burt joined the Committee of the Royal Free Hospital in 1889, and he was elected chairman of the weekly board in 1895, an office which he held for upwards of ten years, to the great advantage of the hospital and of all connected with it. To the chairmanship he added the office of treasurer in 1901, and by his influence and work he did much to save the financial position of the Royal Free Hospital and to place it per-

*Photo.*] [*W. S. Stuart, Richmond.*

Sir Charles Burt, D.L., J.P.

manently on a stronger basis than it had ever before held. Sir Charles was an ideal chairman in every way—firm, courteous, active, knowledgeable, full of initiative and prudent counsel. One of Sir Charles's signal gifts was his power of handling a meeting and getting through business rapidly to the satisfaction of all parties. He deservedly won the confidence of his associates, and exercised a wise and commanding influence in most affairs and institutions with which he became identified.

Sir Charles Burt acted for some time as a visitor for King Edward's Hospital Fund for London. He was also a member of the Central Hospital Council for London, to which he acted as chairman from 1899 to 1905. These were five momentous years for the Council, and under his energetic guidance they proved the most fruitful of any period of its existence. Thus, mainly through his influence, an arrangement was arrived at with the Metropolitan Water Board by which an aggregate saving of about £5,000 per annum was effected for the hospitals. Sir Charles Burt was also instrumental in obtaining the appointment of a Select Committee of the House of Commons on the Exemption of Hospitals from Local Rates, but, despite the exercise of much energy on his part, the Bill introduced to effect this object failed to obtain a second reading. The indebtedness of the hospitals to Sir Charles Burt was, however, expressed by the Council, which passed a hearty vote of thanks to him for the immense trouble he had taken in connection with this question.

Sir Charles Burt belonged to a type of men whom the voluntary hospitals badly need at the present time. He had great driving force, a robust intellect, a sound judgment, and the will to enforce what was right in connection with every business he took in hand. Known in the City as a keen man of business, whose judgment was sound, any appeal from him in support of a particular hospital, or of hospitals generally, was recognised as being one which was entitled to have, and invariably received, the first consideration. Mr. Conrad Thies, the late secretary of the Royal Free Hospital, writes: "Of all the men that I have ever known, Sir Charles Burt most fully carried out the Scriptural injunction. 'Whatsoever thy hand findeth to do, do it with thy might.' Although he has gone from us, his bright example of a useful, strenuous life remains, and his good record ought to inspire many of the present generation to do their duty to their fellow-men with the same singleness of purpose."

## Figures and Tables

**Figure f1:**